# Psychopathological Processes Involved in Social Comparison, Depression, and Envy on Facebook

**DOI:** 10.3389/fpsyg.2018.00022

**Published:** 2018-01-23

**Authors:** Aurel Pera

**Affiliations:** Department of Teacher Training, University of Craiova, Craiova, Romania

**Keywords:** Facebook envy, depressed mood, social comparison, psychological well-being, positive self-presentation

## Abstract

Is Facebook utilization beneficial or detrimental for psychological well-being? I draw on outstanding research (e.g., Chou and Edge, 2012; Lin and Utz, 2015; Appel et al., 2016; Ehrenreich and Underwood, 2016; Vogel and Rose, 2016; Hu et al., 2017) to substantiate that examining other individuals’ positively presented material on Facebook may have detrimental consequences. Increasing comparisons on Facebook may generate feelings of envy, the latter being a significant process determining the effect of growing social comparison on psychological well-being. To date, there is an increasing body of literature investigating the psychological consequences of Facebook usage, the function of relationship closeness in producing the feelings of contentment and envy, the impacts of exposure to positive content on Facebook, the link between envy and depression on Facebook, and the function of tie strength in expecting the emotional results of browsing Facebook. I am specifically interested in how previous research explored the consequences of Facebook use on psychological outcomes, the moderating function of envy in the link between Facebook use and reduced affective wellbeing, the psychological results of non-interactive Facebook conduct, and the role of relationship closeness in anticipating user’s contentment and envy after inspecting a post. A synthesis of the extant literature suggests that inspecting other individuals’ positive news on Facebook brings about contentment through emotional contagion, whereas negative news causes discomfort as a consequence of mood contagion, the transmissible effect being more powerful when the news is associated with a strong tie. The outcomes of this research back the argument that self-confidence and dispositional envy are instrumental in producing Facebook envy. These findings highlight that the emotional results of browsing Facebook are considerably affected by the substance of the comment, the personal attributes of the Facebook user, and link between the reader and the poster. As limitations in the current review, more hypotheses need to be tested and future directions for subsequent multilevel research on the behavioral and cognitive outcomes of Facebook should illuminate why when individuals achieve belongingness demands on Facebook, they feel in a superior way about themselves and their reputation in social circles.

## Introduction

Most recent literature on the consequences of Facebook use on psychological outcomes has insisted upon the substantial link between social comparison, depression, and envy, associated with depressed individuals’ depleted self-confidence and subsequent feelings of mediocrity. Facebook allows individuals to exhibit themselves in an online profile with images and life events aimed for sharing with users registered as their Facebook pals. Facebook users can decide on whom they permit to be Facebook pals, thus implicitly managing the comments, using some heuristics when they conceive impressions of other individuals, particularly people whom they do not know directly. Facebook dispossesses its users from detecting their online pals’ non-verbal expressions, therefore coercing users to depend only on the material they can obtain online. The main goal of this article is to clarify that Facebook social networks develop swifter than concrete social ones, and thus it is quite out of the question for its users to communicate personally with each of their network pals (Chou and Edge, 2012). I aim to prove that, on Facebook, self-presentation is distinctive as users conceive their self-images on openly accessible profiles that can be read by a diversity of other users. Focusing on the processes regulating the co-manifestation of depression and envy, I tackle the literature and develop an account according to which the positive self-presentation predisposition can bring about either positive or negative psychological results for users being determined by whether their Facebook undertakings are other- or self-focused. Such end results vary as diverse mental mechanisms seem to be involved being based on the user’s focus. When the latter homes in on other individuals’ Facebook profiles, positive self-presentation usually makes possible negative results through social comparison (Vogel and Rose, 2016).

## Does the Utilization of Facebook Bring About Feelings of Envy?

A great deal of research has tried to clarify whether envy is significant in the link between Facebook and depressed mood. Appel et al. (2015) suggest that inferior self-confidence in depressed persons brings about increasing social comparison, making envy more likely. Facebook enables effortless impression management, supplying high comparison criteria. Depressed and non-depressed individuals, in a quasi-experimental online research, indicate their self-confidence and are presented with definitely configured Facebook profiles that are either appealing or distasteful. Individuals are required to correlate themselves to the profile owner and to convey their ensuing attitudes of inferiority and envy. Depressed individuals are more envious, particularly after seeing the appealing profile. Envy is related to higher self-reported inferiority and associated adversely with self-confidence. Inferior self-confidence and ensuing attitudes of inferiority are instrumental in depressed persons’ elevated degrees of envy. Lin and Utz (2015) examine the emotional results of reading a comment on Facebook and investigate the function of tie strength in envisaging contentment and envy. Employing a multi-method approach, the authors claim that positive emotions are more dominant than negative ones while surfing Facebook. Tie strength is positively related to the attitude of contentment and harmless envy, whereas malevolent envy excludes tie strength after inspecting a positive comment on Facebook.

Disconnecting the link between social media and depression, Blease (2015) claims that the incompatibility between present social climate and the setting of evolutionary adaption allows expectations concerning the utilization of social media as a determinant for mild depression. Facebook users are more prone to causal drivers for mild depression under such circumstances: (a) the greater the amount of online friends; (b) the greater the time spent inspecting posts from the pool of such individuals; (c) the user does so constantly; and (d) the content of the comments is of a swaggering character. The prevalence and the amount of displays of superior status cues identified by the user induces the perception of inferior relative social relevance among Facebook users. Ehrenreich and Underwood (2016) examine how teenagers’ internalizing symptoms (e.g., depression, restlessness, and alienation) affect the content of their Facebook interaction and the feedback they receive from friends on Facebook. Connections between internalizing symptoms and Facebook interaction are dissimilar for girls and boys. For the former, internalizing symptoms envisage several kinds of Facebook content: detrimental affect, somatic disorders, and eliciting backing – but are not associated with boys’ Facebook posts. In contrast with boys, for girls, internalizing symptoms envisage collecting more peer posts indicating detrimental affect, and peer feedback providing support. Girls predisposed to internalizing symptoms utilize Facebook in manners that are alike to concentrating on adverse attitudes with peers, and receive likely reinforcing responses in return.

Thus, the use of Facebook generates both beneficial and adverse feelings: users may be exposed to comments from a diversity of distinct individuals. The emotional results of inspecting a comment can be determined by the substance of the post and can be affected by the link between the poster and reader. Tie strength between the user and the poster influences emotional results. Inspecting other individuals’ positive news on Facebook brings about contentment through emotional contagion, whereas negative news causes discomfort as a consequence of mood contagion, the transmissible effect being more powerful when the news is associated with a strong tie. Relationship closeness constructively curbs the impact of the substance of Facebook comments on users’ emotions, e.g., reinforcing the feelings of contentment or discomfort (Lin and Utz, 2015).

It follows from the preceding analysis that concentrating on other individuals is constructive when Facebook users examine the profiles of descending comparison targets. The beneficial self-presentation component of Facebook may be favorable when individuals concentrate on themselves when employing Facebook. Concentrating on the self while employing Facebook results in the accomplishment of self-presentation demands and, in the aftermath, of self-affirmation ones. Self-presentation on Facebook is advantageous when the positive self-perception conceived in that respect is employed to enhance and support the self-concept. The self-affirming advantages of concentrating on one’s own profile materialize when the latter comprises positively self-presented material. Individuals who publish chiefly negative material about themselves are improbable to psychologically profit from examining their profiles. Any personal and situational aspects that influence an individual to post negative material would regulate the standard advantages of an emphasis on self-generated material (Vogel and Rose, 2016).

## Envy as Process that May Connect Facebook Utilization and Psychological Well-Being

Consistent research findings in this arena indicate that evaluations of high personal control raise the probability of benign envy compared with malicious envy. Another relevant distinctness between benign and malicious envy refers to the perceived merit of the superior end result of the envied. As Appel et al. (2016) observe, in their Facebook profiles, individuals convey teeming social comparison data disclosing chiefly positive self-portrayals, and thus supplying a productive ground for envy. The authors review research on this mechanism for the possible detrimental consequences of Facebook utilization on well-being and depression. Passive Facebook utilization envisages various measures of social comparison and envy that facilitate a positive link between Facebook utilization and unsatisfactory affective outcomes (e.g., depression). Wallace et al. (2017) note that online social networks (OSNs) provide a flow of information that immediately offers comparison chances, frequently generating feelings of envy. Facebook users undergo greater OSN-situational envy when they display neuroticism and employ Facebook to satisfy needs to collect information, pursue attention, or pass time (envy-prone individuals should utilize OSNs for particular goals and circumvent passive undertakings).

Hu et al. (2017) seek to harmonize research indicating that Facebooking is both behooveful and harmful for individuals’ psychological well-being by elucidating the particular conse quences of Facebooking on persons’ online–offline social relationship contentment and psychological well-being. Employing structural equation modeling, Hu et al. (2017) inspect routes between Facebook strength, online–offline social relationship contentment, perceived social backing, social interplay distress, and psychological well-being, assessing personality dissimilarities on each of the paths. Intensive Facebooking is positively related to individuals’ psychological well-being via online social relationship contentment and adversely associated with their psychological well-being via offline social relationship contentment. The connection between perceived social backing and psychological well-being is more powerful for self-observers than for socializers. The advantages or drawbacks of Facebooking are dependent on both personality attributes and online–offline social settings. Kaya and Bicen (2016) inspect the consequences of Facebook use on students’ behaviors. Whether there is a beneficial link between trust, social media involvement and associated conducts is evaluated with respect to using Facebook. The findings point out that Facebook is utilized for interactive fun and distributing information, pictures and songs. Students are aware that swearing is a type of wrongoing and are cognizant of protecting their social identity. They also adhere to privacy by not using their friends’ Facebook accounts. Ophir et al. (2016) focus on the symptom of youth depression in teenagers’ Facebook status updates, and use a multiple regression analysis to identify status update characteristics that envisage status update depression scores: DSM-5 depressive manifestations, cognitive alterations, poetic-dramatic type of verbal content, and feelings toward other persons.

The argument so far establishes that individuals with high self-confidence obtain substantial social backing on Facebook when posting negative material, whereas individuals with low self-confidence obtain fewer reactions. When individuals post negative material, they are adversely impacted by examining their own profile and by other individuals’ uninspired reactions. Facebook utilization is beneficial when individuals use the advantages of positive self-presentation and undergo positive results, but is detrimental for psychological well-being when individuals examine acquaintances’ positively displayed material, take part in increasing social comparison, and experience negative results. Self-presentation provides a channel through which to examine the conflicting consequences of Facebook use. Concentrating on (non-close) other individuals’ idealistically positive online selves causes negative results, while homing in on their own online selves brings about positive ones (Vogel and Rose, 2016).

Existing research makes the case that tie strength regulates the link between the substance of the comment and the feeling of contentment. Users are content after reading positive comments from their Facebook friends, especially if the good news is associated with a strong tie. Envious feelings are produced by distinct features of the user, e.g., low self-esteem, instead of relationship closeness. For negative news, Facebook users undergo more adverse emotions when it is associated with a strong tie than a weak one. People have to tolerate the negative news associated with their strong ties in real life, because Facebook is not the exclusive communicational medium. A familiar link between the poster and the reader assists in advancing kindliness and the incentive of leveling-up: benign envy takes effect when an envy-inducing comment is displayed by a strong tie instead of a weak tie. Individuals are less expected to undergo malicious envy, a demolishing impetus, in relation to their strong ties in comparison with other less-intimate people (Lin and Utz, 2015).

## Causal Links Between Facebook Utilization, Social Comparison, Envy, and Depression

A robust stream of research has been devoted to understanding envy as a functional emotion indicating the demand to concentrate on perceived dissimilarities in social status provided to the self and others. Chou and Edge (2012) analyze the effect of utilizing Facebook on users’ perceptions of other persons’ lives and assert that those with more profound participation with Facebook have distinct perceptions of other individuals than those less engaged, as Facebook users are likely to build judgment on instances readily recollected (the availability heuristic) and to apply the positive posts displayed on Facebook to other persons’ personality, instead of situational factors (correspondence bias). Individuals who utilize Facebook longer admit more that other persons are more successful, and concede less that life is satisfactory, and individuals spending more time on Facebook weekly recognize more that other persons are more successful and have better lives. Individuals that include more persons whom they do not know in the flesh as their Facebook friends accept more that other people have better lives. Lãzãroiu (2016) insists on the Facebook use intensity, the advancement of Facebook addiction, and the effect of personal features and motivation aspects on Facebook utilization and addictive propensity. The research decodes feasible mechanisms constituting the grounds of maladaptive patterns of Facebook usage, the function of personal psychological characteristics in Facebook use, and significant psychological drivers for Facebook addiction. Bratu (2017) investigates the personality facets and determinants of individuals that engage in Facebook trolling, and identifies the impact of abnormal, disruptive, and non-normative behaviors on online groups, and zappy connections between dark personality traits and socially adverse and malevolent conducts. Morin-Major et al. (2016) analyze the connections between Facebook behaviors (utilization rate, network proportion, self-presentation and peer-interplay) and fundamental degrees of cortisol among teenage boys and girls, incorporating well-substantiated levels of perceived stress, felt social backing, egotism, and depressive syndromes. Cortisol systemic output is beneficially related to the amount of Facebook friends and adversely correlated to Facebook peer-interplay. Facebook behaviors are linked with diurnal cortisol concentrations in teenagers.

Park et al. (2016) explore the social backing underlying forces that typifies the manner persons with fluctuating degrees of depression and Severe Combined Immunodeficiency-diagnosed clinically depressed and non-depressed people network with Facebook. Communicating positive or negative data on Facebook affects the supply of social backing depressed persons (a) really receive (contingent on definite social backing transactions noted down on Facebook walls) and (b) assume they receive (contingent on subjective evaluations) from their Facebook network. Depression is positively associated with social backing from Facebook networks when users communicate negative information. Depression is negatively associated with how much social backing users assume they receive from their Facebook networks. An imbalance typifies the link between depression and various kinds of Facebook social backing. Rosenthal et al. (2016) scrutinize whether adverse Facebook experiences, assessed by kind, newness, amount of experiences, and acuteness of distress, are autonomously related to depressive syndromes among teenagers in a longitudinal family cohort, and identify a coherent link between adverse Facebook experiences and depressive syndromes. Satici and Uysal (2015) employ a stepwise regression analysis with autonomous variables such as life contentment, subjective strength, flourishing, and subjective joy, to clarify fluctuation in problematic Facebook utilization, and indicate that all of them are relevant adverse predictors of such use.

Shakya and Christakis (2017) delve into the links of Facebook activity and actual social network performances with self-reported physical and mental health, self-reported life contentment, and body mass index. The results specify that Facebook use is adversely correlated with well-being, being comparable to or more significant in size than the beneficial effect of offline interactions, thus indicating an attainable balance between offline and online connections. Steers (2016) aims to spell out Facebook’s intricate connection with relatedness demands and depressive syndroms. Facebook is driven by both connection and disconnection, thus impacting individuals’ mental health. Facebook involvement bolstering connection enhances well-being whereas disconnecting undertakings bring about adverse effects. Facebook depression may be grasped by investigating Facebook’s deep-seated characteristics, therefore stimulating and propagating persons’ negative affect. Zhang (2017) examines the impact of self-disclosure on social network sites (SNSs) on young persons’ mental health. The results indicate that individuals are likely to bare their souls on Facebook when they are stressed and that self-disclosure on Facebook facilitates the link between tense life activities and mental health. Facebook disclosure is positively related to displayed social backing on Facebook, generating expanded perceived social support, improved life contentment, and diminished depression.

It thus seems reasonable to claim that self-confidence and dispositional envy are instrumental in producing Facebook envy. Nearly all Facebook comments are not self-evaluation intimidating, and less malicious envy is presumed when it is associated with a close friend. Facebook users with superior dispositional envy are more expected to take part in rising social comparisons and undergo more benign and malicious envy after inspecting a positive comment on Facebook. If Facebook users collect more comments from their close friends, they undergo more contentment. The emotional results of browsing Facebook are considerably affected by the substance of the comment, the personal attributes of the Facebook user, and link between the reader and the poster. Tie strength curbs the feeling of contentment after inspecting a comment on Facebook, in addition to the one of benign envy (Lin and Utz, 2015).

These studies provide evidence that depressiveness is related to superior levels of envy when comparison criteria are high. Facebook use bolsters disapproving social comparisons and envy, possibly generating depressed mood. The idea of depression prompting people to envy assists in determining a community that is particularly exposed to the envy provoking capacity of Facebook comparison. Unbecoming social comparisons brings about two different types of envy. Malicious envy involves the drive to undermine the envied individual, being related to annoyance, reduced personal control, and feelings of unfairness. Similarly irritating, benign envy stimulates to harmonize the superior individual through endeavor and is related to esteem. Disconnecting the two clarifies the contradictory results with regard to Facebook use. Depressed people are mostly predisposed to malicious and not benign envy when using Facebook. Social setting may influence envy on Facebook. The particular link to the comparison criterion shapes the type of envy on Facebook (Appel et al., 2016).

## Conclusion

My findings bring new insights suggesting that examining impeccably elaborate depictions of other individuals’ existences may cause envy, which directs users to assess their own undertakings depressingly and feel unsuccessfully. The main gaps and problems identified by the review cover the aspect that envy on Facebook gives rise to a vicious cycle in which examining idealistically positive profiles results in the production of more sublimely positive material for other individuals to explore. The inferences of the advancements summarized in the previous sections of this article indicate an increasing demand for a research agenda on the tremendously positive character of Facebook material that makes inoffensive interplays seem more intimidating than they under other circumstances would. Preserving an other-focus throughout Facebook undertakings is not all the time detrimental and, under particular situational conditions, concentrating on other individuals is gainful. The rise in self-confidence when examining close friends’ profiles is a result of reflection mechanisms. As limitations in the current review, more hypotheses need to be tested and future directions for subsequent multilevel research on the behavioral and cognitive outcomes of Facebook should illuminate why when individuals achieve belongingness demands on Facebook, they feel in a superior way about themselves and their reputation in social circles (Vogel and Rose, 2016).

## Author Contributions

The author confirms being the sole contributor of this work and approved it for publication.

## Conflict of Interest Statement

The author declares that the research was conducted in the absence of any commercial or financial relationships that could be construed as a potential conflict of interest.
